# Examining if changes in gender-specific and co-ed intramural programs affect youth physical activity over time: a natural experiment evaluation using school- and student-level data from the COMPASS study

**DOI:** 10.1186/s12889-021-12090-z

**Published:** 2021-11-08

**Authors:** Kathleen E. Burns, Ashok Chaurasia, Valerie Carson, Scott T. Leatherdale

**Affiliations:** 1 School of Public Health and Health Systems, University of Waterloo, TJB 2324, 200 University Ave W, Waterloo, ON N2L 3G1 Canada; 2 Faculty of Kinesiology, Sport, and Recreation, University of Alberta, Edmonton, AB Canada

**Keywords:** Intramurals, Co-ed, Gender-specific, Physical activity, Youth, Natural experiment

## Abstract

**Background:**

Despite the benefits of physical activity (PA), the majority of Canadian youth are falling short of the recommended 60 min of moderate-to-vigorous physical activity (MVPA) per day. School-based physical activity programs such as intramurals, are important opportunities for youth to be physically active. There is limited evidence available on the impact of gender-specific (e.g., female-only, male-only) and co-ed intramurals on youth PA over time, however, evidence suggests female-only intramurals may be important for female MVPA. This research aims to capitalize on a natural experiment to generate practice-based evidence on the impact of changes in gender-specific and co-ed intramurals on youth MVPA over time.

**Methods:**

This study used linked longitudinal school- and student-level data from Ontario secondary schools in year 5 (Y5:2016–2017), year 6 (Y6: 2017–2018) and year 7 (Y7: 2018–2019) of the COMPASS study. Data on intramurals from 55 schools were used to determine the changes to gender-specific and co-ed intramurals that occurred from Y5 to Y6. Baseline demographic characteristics were measured and data on PA and sport participation were collected in Y5, Y6 and Y7 on a sample of 4417 students. Hierarchical linear mixed regression models were used to estimate how changes in gender-specific and co-ed intramurals were associated with youth MVPA over time.

**Results:**

Participation in intramural, varsity and community sport were all positively and significantly associated with youth MVPA. Changes in gender-specific and co-ed intramurals were not significantly associated with youth MVPA in Y6 or Y7. However, the positive association between maintaining the addition of gender-specific intramurals on female MVPA in Y7 was on par with the significance level of *α* = 0.05, suggesting that adding gender-specific intramurals may be important for female MVPA.

**Conclusions:**

Adding gender-specific and co-ed intramurals may not be sufficient strategies to increase PA among youth. Further research should explore the effect of gender-specific intramurals on youth PA, as this study may have been underpowered at the school-level. Gender-specific intramurals may promote a supportive PA environment that promotes MVPA, especially among females. These intramurals may be an important component of more comprehensive strategies to increase youth MVPA.

## Background

Moderate-to-vigorous physical activity (MVPA) is important for physical and mental health among youth and is critical for healthy development and disease prevention [1, 2]. Despite the multitude of benefits, approximately two thirds of Canadian youth are falling short of the recommended 60 min of MVPA per day [3–5]. Youth PA also tends to decrease with age [6, 7], with large declines observed during secondary school when sedentary behaviours typically increase [6–9] and the participation in physical education [10, 11] and sports [12, 13] typically decreases. These trends in youth PA are alarming because physical inactivity is a leading modifiable risk factor for premature death [14–16]. Youth are an important target population for PA interventions because health behaviours, such as PA, continue to develop during this time [8, 17–19]. Modest increases in PA can have a positive impact on health and risk of disease [20], and school-level PA interventions can encourage these modest increases and effectively target physical inactivity among youth [21–23].

School-based PA programs are effective strategies to increase PA among youth, because they can effectively target and reach youth populations while reducing common barriers to youth PA (e.g., time, transportation, skill/ability) [24–26]. School-based PA programs are especially effective when they are perceived as inclusive and accessible by students [27]. Intramurals are activities that are participated in by students within the same school, and are an example of such inclusive and accessible programs, as they generally do not require a high-level of skill or a fee to participate in [28]. Intramurals are considered inclusive and accessible, especially compared to other school-based programs (e.g., varsity sports), which typically require a higher level of skill and involve competition against students from other schools [28]. The positive association between participation in intramurals and PA among youth has been well-documented [21, 29–31], and their impact on youth MVPA over time has been previously explored [32, 33]. Specifically, previous studies have explored how real-world changes in intramurals affect youth MVPA over time, and although general changes in intramurals were not associated with youth MVPA over time [33], adding a combination of individual (e.g., running) and team (e.g., soccer) intramurals was positively associated with female MVPA [32]. Our earlier evidence suggested that adding a variety of intramurals had a positive effect on female MVPA regardless of intramural participation, highlighting the positive and indirect relationship between changes in intramurals and MVPA [32]. In addition to individual and team intramurals, intramurals are further categorized by gender-specific (e.g., female-only, male-only) and co-ed, which may have differential effects on the PA of female and male youth. Limited research has been done to examine how gender-specific and co-ed intramurals associate with PA; however, previous research suggests that gender-specific intramurals may be an important factor for intramural participation, particularly among female students. For example, a previous study found that female students attending schools with female-only intramurals were more likely to participate in intramurals compared to females attending schools without these female-only intramurals, and no such association was observed for males [34]. Female youth consistently achieve less PA compared to their male counterparts [3–5], suggesting that gender-specific sports may be an important opportunity for female PA.

There is limited research available on the impact of gender-specific and co-ed intramurals on youth MVPA over time. Given the previous positive relationship observed between adding individual and team intramurals and female MVPA [32], a practical next step would be to examine how gender-specific and co-ed intramurals affect youth MVPA. Schools continue to make changes to gender-specific and co-ed intramurals each year, and these changes are considered a natural experiment because they are not under control of the researchers [35]. These changes in school-level intramurals create the unique opportunity to evaluate a natural experiment and generate practice-based evidence [36]. Therefore, the objective of this study is to use a natural experimental study design to evaluate how changes in school-based gender-specific and co-ed intramurals are associated with female and male youth MVPA over time.

## Materials and methods

### Host study

The COMPASS study is an ongoing 9-year prospective cohort study (2012–2021) collecting hierarchical and longitudinal data from a convenience sample of secondary school students in grades 9–12 and the schools they attend in Alberta, British Columbia, Ontario and Quebec [37]. The COMPASS study used active-information, passive-consent parent/guardian permission protocols, and active student assent, where students could refuse to participate at any time. Participating students completed the COMPASS student questionnaire (Cq), which is a paper-based, self-administered, anonymous survey administered annually during class time. Each year, senior administration (e.g., teachers, principals, administrative staff) at each participating school completed the School Policies and Practice (SPP) questionnaire online. Details on the COMPASS host study, including sampling and the Cq and SPP data collection tools, are available online (www.compass.uwaterloo.ca). The COMPASS study was approved by the Human Research Ethics Board at the University of Waterloo (ORE 30118) and appropriate school board and school committees.

### Study design

This study used school-and student-level data from the COMPASS host study and builds on our previous research examining intramural changes and youth MVPA [33, 38]. Students who were in grade 9 (13–14 years old) and 10 (14–15 years old) at the baseline time point for this study (Y5), with linked data across all three years were included in the study. A total of 5514 students from the 55 Ontario schools were linked over the three-year study period. Main reasons for non-linkage were students transferring schools, students not providing data on grade in Y5 or Y6, students who were absent or had a spare period during the time of Y5 or Y6 data collection, those who left secondary school early, or inaccurate data provided to link measures on the Cq. Details on the methods of COMPASS data linkage are available elsewhere [39, 40]. Only students with: i) complete data on all covariates and ii) complete data or monotone missingness on the outcome were included in the analysis for a final sample size of 4417 students.

This study used a longitudinal quasi-experimental study design, where data on the outcome were measured at pre-intervention (Y5), intervention (Y6) and post-intervention (Y7) time points and were compared between non-randomized intervention and control groups [36]. In natural experimental studies, this design is considered the gold standard research methodology, and important school- and student-level covariates were measured and controlled for through stratification and adjustment to mitigate bias due to confounding from lack of randomization [36].

### Participants

This study utilized COMPASS host study data from 55 Ontario schools that participated in Y5, Y6 and Y7 of the COMPASS study. Schools in other provinces were excluded from this study because of small sample sizes at baseline (Y5) and provincial differences in PA programs and policies. Ontario schools (*n* = 36) were excluded if they did not participate across all three years.

### Measures

#### Outcome

The Cq was used to measure average daily MVPA for students in Y5, Y6 and Y7. The Cq asks students to record their daily time (hours and/or minutes) spent engaging in hard and moderate PA for the last 7 days (e.g., Monday-Sunday) by the following two prompts:  (1) “Mark how many minutes of HARD PA you did on each of the last 7 days. This includes PA during physical education class, lunch, after school, evenings, and spare time” and (2)“Mark how many minutes of MODERATE PA you did on each of the last 7 days. This includes PA during physical education class, lunch, after school, evenings, and spare time. Do not include time spent doing hard physical activities”. To aid with recall, the Cq provides students with descriptions of moderate and vigorous activities. Moderate physical activities are described as “lower intensity activities such as walking, biking to school, and recreational swimming”, and vigorous physical activities are described as “physical activities include jogging, team sports, fast dancing, jump-rope, and any other physical activities that increase your heart rate and make you breath hard and sweat”. These descriptions align with the Canadian Society for Exercise Physiology definitions that are used in PA research among youth [41]. Average daily MVPA is derived by summing the total time in minutes of moderate PA and hard (vigorous) PA for each day (Monday-Sunday) and dividing this sum by 7 days. This self-reported measure of MVPA on the Cq has demonstrated satisfactory reliability and validity, making it an acceptable measure of MVPA in school-based research [42, 43].

#### School-level predictor: changes in gender-specific and co-ed intramurals

In line with our previous research [33, 38], changes in gender-specific and co-ed intramurals were measured in the Ontario schools by comparing intramural data from Y5 to Y6. School administrators were asked in Y5 and Y6 to select the intramural programs/club activities involving PA that were offered to students at their school over the past 12 months. The intramural selections include a variety of activities (e.g., fitness club, rock climbing, soccer, baseball) and include spaces for unlisted activities. School administrators were also asked to indicate whether the intramural offerings were for females only, males only, or co-ed. Changes in gender-specific and co-ed intramurals from Y5 to Y6 were then determined by comparing intramural data between these years. Schools made many changes to intramurals from Y5 to Y6, and these changes were classified into one of the following four groups based on the quantity of gender-specific and co-ed intramurals: (1) schools that primarily added co-ed intramurals from Y5 to Y6, (2) schools that added gender specific intramurals from Y5 to Y6, (3) schools that added and removed the same number of female-only, male-only or co-ed intramurals from Y5 to Y6, and (4) schools that primarily removed female-only, male-only or co-ed intramurals from Y5 to Y6 (reference). These groups were coded and simplified into the following four categories: (1) primarily added co-ed, (2) added gender-specific, (3) no net change, and (4) removed intramurals (reference). Schools classified as: (2) added gender-specific, are schools that either: (i) added the same number of male- and female-only intramurals from Y5 to Y6, (ii) schools that primarily added female-only intramurals from Y5 to Y6, or (iii) schools that primarily added male-only intramurals from Y5 to Y6. Schools classified as: (3) no net change, are schools that removed a gender-specific or co-ed intramural for every one they added (e.g., added one co-ed, removed one co-ed). Schools classified as: (1) primarily added co-ed intramurals, (2) added gender-specific programs, and (3) no net change in intramurals, were the non-randomized intervention groups and the non-randomized comparison group were schools classified as (4) removed intramurals (reference).

#### Student-level correlates

Consistent with our previous research [33, 38], data on student-level sport participation and sociodemographic factors that were known to associate with the predictor and/or outcome were measured by the Cq and included in the analyses. These variables include intramural sport participation, varsity sport participation, community and sport participation. Intramural sport participation, varsity sport participation and community sport participation were measured at Y5, Y6 and Y7. *Intramural sport participation* is measured by asking “Do you participate in before-school, noon hour, or after school physical activities organized by your school? (e.g., intramurals, non-competitive clubs)”, with the response options of “No” (Reference) and “Yes”. *Varsity sport participation* is measured by asking “Do you participate in competitive school sports teams that compete against other schools? (e.g., junior varsity or varsity sports)”, with the response options of “No” (Reference) and “Yes”. *Community sport participation* is measured by asking “Do you participate in league or team sports outside of school?”, followed by the response options of “No” (Reference) and “Yes”. Demographic data were measured at baseline using the Cq and for the following variables with the corresponding response options in brackets: *gender* [female (ref), male], *ethnicity* [White, Black, Asian, Indigenous (First Nations, Métis, Inuit) Latin American/Hispanic, and Other)] and *weekly spending money* [$0, $1 to $5, $6 to $10, $11 to $20, $21 to $40, $41 to $100, more than $100, I do not know]. *Ethnicity* and *weekly spending money* were collapsed into the following categories to ensure adequate cell count: *ethnicity* [white (ref), other]*, weekly spending money* [Zero (ref), $1–$20, $21–$100 and $100+].

#### School-level correlates

Important school-level correlates associated with the predictor and/or outcome were measured and included in the analyses, and include the number of intramural programs in Y5, school size, changes in other PA programs and school neighbourhood median income [33, 38]. The *number of intramural programs in Y5* and *school size* are both continuous variables measured at baseline using the SPP. Data on other school-based PA programs that may affect student MVPA and/or intramural participation (e.g., school PA events) were obtained from the SPP and compared between Y5 and Y6 to derive the variable *changes in PA programs from Y5 to Y6.* This variable was used to categorize school as either: “no change in PA programs”, “added PA programs” or “removed PA programs”. *School neighbourhood median income* was obtained from the 2016 Canadian Census and are based on the median income of the area surrounding the school at baseline [44].

### Analyses

The analytical methods employed in this research are consistent with our previous studies that examined how changes in intramurals associate with youth MVPA over time [33, 38]. All analyses were performed in SAS 9.4. Descriptive analyses were conducted on school- (*N* = 55) and student-level (*N*=4417) characteristics. Summary statistics of individual variables were reported by frequency and percentage for class/categorical variables, while means and standard deviation were used for continuous/discrete variables. Exploratory differences between female and male students on student-level characteristics at baseline were examined using Chi-Square. The unconditional means model (i.e., linear mixed model with only a random intercept and not predictors) stratified by gender was used to estimate the intraclass correlation (ICC) to determine the within-school variation in MVPA. Linear mixed models stratified by gender were used to estimate how changes in gender-specific and co-ed intramurals in Y5 to Y6 were associated with MVPA over time. These models were hierarchical to account for clustering of students within schools and students over time and controlled for relevant student (grade, ethnicity, weekly spending money, intramural sport participation, varsity sport participation, community sport participation) and school (changes in PA programs, number of intramurals in Y5, school size, and school neighbourhood median income) factors. Two indicator variables were created to represent the yearly change in gender-specific and co-ed intramurals: (i) gender-specific and co-ed intramural change in Y6 and (ii) gender-specific and co-ed intramural change in Y7. These indicator variables were included in the models and allowed for the assessment of their effect on MVPA at the intervention year (Y6) and post-intervention year (Y7). For Y7, the effect of changes in gender-specific and co-ed intramurals was assessed under the assumption that changes from Y6 would continue into Y7. This assumption to suppose the changes from Y5 to Y6 onto Y7 was reasonable to meet the objective of this study, which is to evaluate how changes in schools’ gender-specific and co-ed intramural offerings from Y5 to Y6 were associated with youth MVPA over time (e.g., into Y6 and Y7).

## Results

### School-level descriptive characteristics

Statistics describing the characteristics of the school-level sample are presented in Table 1. Specific to the changes in gender-specific and co-ed intramurals from Y5 to Y6, 27 schools primarily added co-ed intramurals, 8 added gender-specific intramurals, 3 schools made no net change to intramurals, and 17 school removed intramurals. Five schools reported adding PA programs from Y5 to Y6. In Y5, the mean school neighbourhood median income was $69,804 (SD = $15,404) and the mean school size was 669 students (SD = 288). An average of 5.4 (SD = 4.1) intramural programs were offered at the schools in Y5.

**Table 1:** Descriptive Statistics for School-Level Characteristics for the sample (*n* = 55) from Year 5 and 6 (2016–2017) of the COMPASS Study

Variable		Freq	%
Changes in Types of Intramurals from Y5 to Y6	Primarily Added Co-ed Intramurals	27	49.1
	Added Gender-Specific Intramurals	8	14.6
	No Net Change in Intramurals	3	5.5
	Removed Intramurals (Reference)	17	30.9
Changes in Other PA Programs from Y5 to Y6	No Change (Ref)	50	90.9
	Added Programs	5	9.1
	Removed Programs	0	0
Variable		Mean	SD
School Neighbourhood Median Income in Y5		$69, 804	$15,404 Min: $31,763 Max: $107, 702
School Size In Y5		669	288 Min: 136 Max: 1550
Number of Intramurals Offered in Y5		5.4	4.1 Min: 0 Max: 14

*Percent values may not sum to 100 due to rounding*

### Student-level descriptive characteristics

Statistics describing the baseline characteristics and the time-varying characteristics of the student-level sample are presented in Table 2 and Table 3. As shown in Table 2, 54% (*n* = 2402) of the sample were female, 73% (*n* = 3210) were white and $1–$20 was most frequently (43%, *n* = 1875) reported amount of weekly spending money. Presented in Table 3, intramural participation among female students was 38% in Y5, 36% in Y6 and 33% in Y7 and 39% in Y5, 37% in Y6 and 36% in Y7 among male students. Average daily MVPA decreased among female and male students over time, with females reporting 105 min (SD = 66) in Y5, 97 min (SD = 64) in Y6 and 89 min (SD = 61) in Y7 and males reporting an average of 117 min (SD = 68) in Y5, 109 min (SD = 68) in Y6 and 102 min (SD = 65) in Y7.

**Table 2:** Descriptive Statistics for Baseline Student-Level Characteristics for the sample (n = 4417) from Year 5 (2016–2017) of the COMPASS study

Variable		Total *n* = 4417	Female (Ref) *n* = 2402 (54%)	Male *n* = 2015 (46%)			
		Freq (%)	Freq (%)	Freq (%)	DF	Chi-Square	P-Value
Grade	Grade 9 (Ref)	2434 (55.1)	1335 (55.6)	1099 (54.5)	1	1.431	0.232
	Grade 10	1983 (44.9)	1067 (44.4)	916 (45.5)			
Ethnicity	White (Ref)	3210 (72.7)	1744 (72.6)	1466 (72.8)	1	0.036	0.849
	Other	1207 (27.3)	658 (27.4)	549 (27.2)			
Weekly Spending Money	Zero (Ref)	1130 (25.6)	541 (22.5)	589 (29.2)	3	107.400	<.0001
	$1–$20	1875 (42.5)	1062 (44.2)	813 (40.3)			
	$21–$100	1065 (24.1)	628 (26.1)	437 (21.7)			
	$100+	347 (7.9)	171 (7.1)	176 (8.7)			

*Percent values may not sum to 100 due to rounding*

**Table 3:** Descriptive Statistics for Time-Varying Student-Level Characteristics for the sample (n = 4417) from Year 5 (2016–2017), Y6 (2017–2018) and Y7 (2018–2019) of the COMPASS study

Variable		Total n = 4417			Female (Ref) n = 2402			Male n = 2015		
		Year 5	Year 6	Year 7	Year 5	Year 6	Year 7	Year 5	Year 6	Year 7
		Freq (%)	Freq (%)	Freq (%)	Freq (%)	Freq (%)	Freq (%)	Freq (%)	Freq (%)	Freq (%)
Intramurals	No (Ref)	2733 (61.9)	2813 (63.7)	2903 (65.7)	1494 (62.2)	1542 (64.2)	1616 (67.3)	1239 (61.5)	1271 (63.1)	1287 (63.9)
	Yes	1684 (38.1)	1604 (36.3)	1514 (34.3)	908 (37.8)	860 (35.8)	786 (32.7)	776 (38.5)	744 (36.9)	728 (36.1)
Varsity	No (Ref)	2568 (58.1)	2529 (57.3)	2688 (60.9)	1464 (60.9)	1447 (60.2)	1558 (64.9)	1104 (54.8)	1082 (53.7)	1130 (56.1)
	Yes	1849 (41.9)	1888 (42.7)	1729 (39.1)	938 (39.1)	955 (39.8)	844 (35.1)	911 (45.2)	933 (46.3)	885 (43.9)
Community	No (Ref)	2109 (47.7)	2394 (54.2)	2781 (63.0)	1218 (50.7)	1377 (57.3)	1599 (66.6)	891 (44.2)	1017 (50.5)	1182 (58.7)
	Yes	2308 (52.3)	2023 (45.8)	1636 (37.0)	1184 (49.3)	1025 (42.7)	803 (33.4)	1124 (55.8)	998 (49.5)	833 (41.3)
Variable		Mean (SD)	Mean (SD)	Mean (SD)	Mean (SD)	Mean (SD)	Mean (SD)	Mean (SD)	Mean (SD)	Mean (SD)
MVPA (min/day)*		110 (67) n = 4417	102 (66) *n* = 4414	95 (64) *n* = 4375	105 (66) n = 2402	97 (64) *n* = 2400	89 (61) *n* = 2380	117 (68) n = 2015	109 (68) *n* = 2014	102 (65) *n* = 1995

*Note that the sample sizes for MVPA in Y6 and Y7 are different compared to baseline as well as Y6 and Y7 of other covariates. This is because some subjects included in the model are missing MVPA data in Y6 and Y7 (i.e., monotone pattern)

### Results from linear mixed models

The ICC was used to estimate the variability in MVPA among female and male students that were attributed to between-school differences. Between-school differences accounted for 1.91% of the variability in female MVPA and 2.09% in male MVPA, which suggests that the characteristics of the school a female or male student attends, are modestly associated with their MVPA. Results from the linear mixed models are presented in Table 4. Female and male students in grade 10 at baseline accumulated significantly less average MVPA minutes per day compared to those in grade 9 (females: $\hat{\beta}=$-8.428, *p* < 0.0001, males: $\hat{\beta}=$-7.298, *p* = 0.001). MVPA decreased over time for both females and males, although this was only significant for female students (females: $\hat{\beta}=-7.253$, *p* = 0.004, males $\hat{\beta}=$-1.100, *p* = 0.692). Sport participation was positively associated with female and male MVPA, as students participating in school-based intramurals (female: $\hat{\beta}=$ 4.998, *p* = 0.003, male: $\hat{\beta}=$ 9.728, *p* < 0.0001), school-based varsity sports (female: $\hat{\beta}=$ 16.161, p < 0.0001, male: $\hat{\beta}=$ 18.003, p < 0.0001) and community sports (female: $\hat{\beta}=$ 26.084, p < 0.0001, male: $\hat{\beta}=$ 20.310, p < 0.0001) achieved significantly more average MVPA minutes per day, all compared to those who reported no participation.

**Table 4:** Linear Mixed Models examining the association between changes in intramural programming in Y6 on MVPA in Y6 and Y7 of the COMPASS Study stratified by gender

Variable		Female *n* = 2402			Male *n* = 2015		
		Estimate	95% CI	p-value	Estimate	95% CI	p-value
Effect of Intramural Change on MVPA in Y6	Removed Intramurals (Reference)	–		–	–	–	–
	Primarily Added Co-ed Intramurals	4.695	−0.699-10.089	0.088	−1.163	−7.32-4.998	0.711
	Added Gender-Specific Intramurals	5.098	−2.382-12.578	0.182	−4.703	−13.194-3.787	0.278
	No Net Change in Intramurals	2.236	−4.060-8.534	0.486	5.148	−1.780-12.075	0.145
Effect of Intramural Change on MVPA in Y7	Removed Intramurals (Reference)	–	–	–	–	–	–
	Primarily Added Co-ed Intramurals	1.749	−3.66-7.159	0.526	0.411	−5.764-6.587	0.896
	Added Gender-Specific Intramurals	7.507	−0.0126-15.027	0.050	−3.764	−12.276-4.748	0.692
	No Net Change in Intramurals	−1.502	−12.079-9.075	0.781	8.747	−2.818-20.312	0.138
Grade	Grade 9 (Ref)	–	–	–	–	–	–
	Grade 10	−8.428	−12.131--4.724	**<.0001**	−7.298	−11.421--3.175	**<.0001**
Year		−7.253	−12.391--2.348	**0.004**	−1.100	−6.554-4.353	0.692
Intramural Sport Participation	No (Ref)	–	–	–	–	–	–
	Yes	4.998	1.7647–8.2318	**0.003**	9.728	6.051–13.406	**<.0001**
Varsity Sport Participation	No (Ref)	–		–			–
	Yes	16.161	12.506–19.815	**<.0001**	18.003	13.902–22.104	**<.0001**
Community Sport Participation	No (Ref)	–		–			–
	Yes	26.084	22.784–29.384	**<.0001**	20.310	16.520–24.101	**<.0001**

*Models controlled for changes in PA programs in Y6, median school neighbourhood income in Y5, school enrolment in Y5, number of intramurals in Y5, ethnicity and weekly spending money. Values significant at α*= *0.05 are bolded*

The associations between changes in gender-specific and co-ed intramurals and MVPA were non-significant for both female and male youth, regardless of whether students reported participating in these programs. Specifically, primarily adding co-ed intramurals, adding gender-specific intramurals and making no net changes to intramurals in Y6 were all positively, but non-significantly associated with female MVPA in Y6. If these changes were to continue into Y7, primarily adding co-ed intramurals and gender-specific intramurals were both positively, but non-significantly associated with female MVPA one year later, into Y7. This significance of the association between maintaining the addition of gender-specific intramurals on female MVPA in Y7 was on par with significance level of *α* = 0.05; and hence was inconclusive. If schools that added gender-specific intramurals in Y6 maintained these changes into Y7, female students accumulated an average of 7.507 more daily minutes of MVPA compared to female students attending schools that removed intramurals. Lastly, if schools maintained no net changes from Y6 into Y7, this was negatively and non-significantly associated with female MVPA in Y7.

Primarily adding co-ed intramurals and gender specific intramurals in Y6 was negatively and non-significantly associated with male MVPA in Y6. If these changes were to continue into Y7, primarily adding co-ed intramurals was positively but non-significantly associated with male MVPA into Y7, while adding gender-specific intramurals was negatively and non-significantly associated with male MVPA in Y7. No net changes in intramurals in Y6 was positively and non-significantly associated with male MVPA in Y6. If these changes were maintained in Y7, the direction of this association was consistent, as no net changes was positively and non-significantly associated with male MVPA in Y7 as well.

## Discussion

To our knowledge, this was the first study to evaluate how changes in gender-specific and co-ed intramurals were associated with MVPA over time. This study builds on previous research [32, 33] using COMPASS data which found that although general changes to intramurals were unrelated to youth MVPA, adding a combination of individual and team intramurals had a positive impact on female MVPA. Considering that intramurals are also divided into gender-specific and co-ed intramurals, exploring the association between these specific intramurals and MVPA among youth was a logical progression of this previous research. This association was examined using a large, linked longitudinal school- and student-level dataset, and employed an innovative methodology that allowed for the examination of the effect of changes in gender-specific and co-ed intramurals on MVPA in Y6 and into Y7, under the assumption that these changes were continued from Y6. The results of this study suggest that youth MVPA generally decreases over time, and although no statistically significant associations were observed, the effect of gender-specific intramurals on female MVPA may be important, and should be further explored. Future research should explore the associations between gender-specific intramurals and youth MVPA over time using a larger school sample to ensure adequate power at the school-level.

As expected, [3, 4, 45, 46] average daily MVPA decreased among female and male students over time, and female and male students in grade 10 at baseline achieved significantly less daily MVPA minutes per day compared to youth in grade 9 at baseline. The decreasing trends in MVPA over time among youth may be attributed to many social, behavioural and environmental factors typically observed during this time which may include perceived lack of time [47], decreases in sport participation [12, 13], and increased screen time and sedentary behaviour [6–9]. Although MVPA declined over time for both females and males, this association was only significant for females, which may be explained by the greater decrease in MVPA over time typically observed in females compared to males [48, 49]. Sport participation was positively associated with MVPA among female and male youth. Other research supports these findings, as intramural [21, 30, 31], varsity [30, 31] and community [30, 50–52] sport participation all positively associate with MVPA among female and male youth. These school- and community-based sports provide opportunities for PA, which is an important correlate of youth MVPA [26, 52, 53].

Primarily adding co-ed intramurals and no net change in intramurals were not associated with female or male MVPA in Y6 or Y7. Although no research was found directly on intramural change, other research on intramurals supports this finding, as the availability of intramurals was not directly associated with student MVPA [23, 29]. Although the environmental context can influence youth MVPA, intrapersonal and interpersonal characteristics may be more important predictors of MVPA [54], which may help explain these nonsignificant findings. For example, intrapersonal factors such as self-efficacy and enjoyment, and interpersonal factors such as peer support for PA are all positively associated with youth MVPA [54–57], and aspects of school-PA environment, such as the availability of school programs [54], may not be as important for youth MVPA. Therefore, changes in the school environment, such as adding co-ed intramurals, or making no net changes to intramurals, may not be sufficient approaches to increasing MVPA among youth. More comprehensive strategies to address the physical inactivity among youth are warranted. For example, PA programs that include a combination of changes to the school environment (e.g., school curriculum, school-based policies), community engagement and parental involvement have proven effective in improving youth PA [24, 58, 59] and warrant further investigation and evaluation.

There were no significant associations between adding gender-specific intramurals and MVPA among female or male students. However, there may not have been adequate power at the school-level to detect a significant association, as only 8 schools reported adding gender-specific intramurals in our sample. Although this association may be underpowered at the school-level, it is important to note that adding gender-specific intramurals was positively associated with female MVPA in Y7, and the *p*-value of this association warrants further exploration using a larger school-level sample. Past research has suggested that gender-specific intramurals are more important for female youth participation, compared to males [34]. Females may face more barriers to sport participation and PA compared to males, and these barriers range from intrapersonal to environmental factors, as described by the social-ecological model [60, 61]. More specifically, females may face intrapersonal barriers (e.g., low self-efficacy), interpersonal barriers (e.g., time-restraints and lack of social support from peers), and environmental barriers (e.g., lack of choice and limited female-only opportunities in PA programs) [61]. To address these barriers, strategies to increase self-efficacy, provide accessible PA programs and create a supportive PA environment are warranted [61]. Gender-specific intramurals may provide females with this environment by offering supportive and accessible opportunities to be physically active, and reducing some of these barriers to PA. For example, female-only intramurals may increase confidence and self-efficacy by providing a safe space to participate in PA, that is free of intimidation or excessive competition that may be present in a co-ed environment [21, 61]. These female-only programs may also address time-restraints by providing access to PA during school-time and may promote positive interactions with peers and encourage peer support for PA [54–57]. Finally, gender-specific intramurals address programming barriers, by providing a variety of female-only opportunities to be physically active [61].

### Strengths and limitations

This research comprehensively examined how changes in gender-specific and co-ed intramurals were associated with youth MVPA over time and addressed an important gap in the literature to expand on our understanding of how changes to school-level intramurals affect youth PA outcomes. This study utilized a large linked sample of longitudinal student- and school-level data, and employed a robust evaluation of a natural experiment to generate practice-based evidence on school intramurals. Additionally, this study utilized a novel approach to program evaluation through the use of indicator variables, and future research may consider using such methods to evaluate programs and generate timely public health evidence.

It is also important to note the limitations of this study. Firstly, the generalizability of these results may be limited, as COMPASS uses convenience sampling to recruit schools. However, the COMPASS study has a large sample size and employs an active-information, passive-consent procedure to promote participation and honest responses from students [62]. Additionally, this procedure may produce more robust results by limiting self-selection and response biases [37]. Secondly, because schools made many changes to their gender-specific and co-ed intramurals, the intervention groups may have been diluted. For example, the intervention groups consisted of schools that: (i) “primarily” added co-ed intramurals, (ii) “primarily” added gender-specific and (iii) no net changes to intramurals, as opposed to schools that: (i) “only” added co-ed intramurals, (ii) “only” added gender-specific intramurals and (iii) no changes to intramurals. These diluted intervention groups may have made associations difficult to detect, which highlights the complexity of evaluating PA programs using a natural experiment. Thirdly, this study may have been under-powered at the school-level, possibly limiting the ability to detect associations between changes in gender-specific intramurals on youth MVPA over time. However, this study provides insight into the potential role of gender-specific and co-ed intramurals on youth MVPA, and future research should explore these associations using a larger school-level sample to ensure adequate power.

## Conclusions

This study found that intramural and varsity sports are important school-based opportunities for youth PA, as participation in these sports were positively associated with MVPA. To encourage sport participation and PA among students, schools should continue to offer a variety of intramural and varsity sports. Changes in gender-specific and co-ed intramurals were not associated with female or male MVPA over time. Changes in the school environment, such as adding gender-specific and co-ed intramurals may not be sufficient strategies to increase PA among youth, although further research should explore the effect of gender-specific intramurals on youth PA, as this study may have been underpowered at the school-level. Gender-specific intramurals may promote a supportive PA environment by fostering self-efficacy, enjoyment and peer support, especially among females. These intramurals may be an important component of more comprehensive strategies to increase youth PA.

## Data Availability

COMPASS study data is available upon request through completion and approval of an online form: https://uwaterloo.ca/compass-system/information-researchers/data-usage-applicationThe datasets used during the current study are available from the corresponding author on reasonable request.

## Abbreviations

COMPASS: The Cannabis use, Obesity, Mental health, PA, Alcohol use, Smoking and Sedentary behaviour study
MVPA: Moderate-to-vigorous PA
ICC: Intraclass Correlation
PA: Physical Activity
